# Developing Virtual Reality Head Mounted Display (HMD) Set-Up for Thoracoscopic Surgery of Complex Congenital Lung MalFormations in Children

**DOI:** 10.3390/children9010050

**Published:** 2022-01-03

**Authors:** Gloria Pelizzo, Sara Costanzo, Margherita Roveri, Giulia Lanfranchi, Maurizio Vertemati, Paolo Milani, Gianvincenzo Zuccotti, Simone Cassin, Sebastiano Panfili, Francesco Rizzetto, Alessandro Campari, Anna Camporesi, Valeria Calcaterra

**Affiliations:** 1 Pediatric Surgery Department, “Vittore Buzzi” Children’s Hospital, 20154 Milan, Italy; sara.costanzo@asst-fbf-sacco.it (S.C.); margherita.roveri@asst-fbf-sacco.it (M.R.); giulia.lanfranchi@asst-fbf-sacco.it (G.L.); 2 Department of Biomedical and Clinical Science “Luigi Sacco”, University of Milan, 20157 Milan, Italy; maurizio.vertemati@unimi.it (M.V.); gianvincenzo.zuccotti@unimi.it (G.Z.); simone.cassin@unimi.it (S.C.); sebastiano.panfili@unimi.it (S.P.); 3 CIMaINa (Interdisciplinary Centre for Nanostructured Materials and Interfaces), University of Milano, 20133 Milan, Italy; paolo.milani@unimi.it; 4 Department of Physics “Aldo Pontremoli”, University of Milano, 20157 Milan, Italy; 5 Pediatric Department, Children’s Hospital “Vittore Buzzi”, 20154 Milan, Italy; valeria.calcaterra@unipv.it; 6 Department of Radiology, ASST Grande Ospedale Metropolitano Niguarda, Piazza Ospedale Maggiore 3, 20162 Milan, Italy; francesco.rizzetto@unimi.it; 7 Postgraduate School of Diagnostic and Interventional Radiology, University of Milano, 20157 Milan, Italy; 8 Pediatric Radiology and Neuroradiology Unit, “Vittore Buzzi” Children’s Hospital, 20154 Milan, Italy; alessandro.campari@asst-fbf-sacco.it; 9 Division of Pediatric Anesthesia and Intensive Care Unit, Department of Pediatrics, Children’s Hospital Vittore Buzzi, 20154 Milan, Italy; anna.camporesi@asst-fbf-sacco.it; 10 Pediatrics and Adolescentology Unit, Department of Internal Medicine, University of Pavia, 27100 Pavia, Italy

**Keywords:** virtual reality, pediatric surgery, congenital lung malformation, children, thoracoscopic surgery

## Abstract

Video assisted thoracoscopic surgery (VATS) has been adopted in pediatric age for the treatment of congenital lung malformations (CLM). The success of VATS in pediatrics largely depends on the surgeon’s skill ability to understand the airways, vascular system and lung parenchyma anatomy in CLM. In the last years, virtual reality (VR) and 3-dimensional (3D) printing of organ models and VR head mounted display (HMD) technologies have been introduced for completion of preoperative planning in adult patients. To date no reports about the use of VR HMD technologies in a pediatric setting are available. The aim of this report is to introduce a VR HMD model in VATS procedure to improve the quality of care in children with CLM. VR HMD set-up for planning thoracoscopic surgery was performed in a series of pediatric patients with diagnosis of CLM. The preoperative VR HMD evaluation allowed a navigation into the malformation with the aim to explore, interact, and make the surgeon more confident and skilled to answer to the traps. A development of surgical simulations models and teaching program dedicated to education and training in pediatric VATS is suitable among the pediatric surgery community. Further studies should demonstrate all the benefits of such technology in pediatric patients submitted to VATS procedure.

## 1. Introduction

Congenital lung malformations (CLM) represent a wide range of disorders that include congenital cystic adenomatoid malformation, intra and extra-lobar pulmonary sequestration, bronchogenic cysts, congenital lobar emphysema and bronchial atresia [1,2,3]. During lung development, the cellular crosstalk can be altered or interrupted, leading to the impairment of lung branching and alveolar formation, resulting in the malformation [4,5,6,7,8].

CLM have a cumulative incidence of 30–42 cases per 100,000 individuals and they account for 5–18% of all congenital abnormalities; however, the prevalence may be underestimated because an unknown proportion of CLM is postnatally detected by chance or because of unexplained recurrent or persistent respiratory symptoms or signs [9]. At birth, CLM may present with respiratory distress necessitating prompt diagnosis and treatment.

In the last years, virtual reality (VR) and 3-dimensional (3D) printing of organ models and VR head mounted display (HMD) technologies have been introduced for completion of preoperative planning in adult patients [10,11]. The ability to simultaneously assess cardiovascular and airway components increases the understanding of conditions referred to anatomical anomalies and allows anticipation of potential surgical difficulties in the treatment of adult patients affected by cardiothoracic disease. Moreover, 3D reconstructions of pulmonary anatomy for a preoperative planning in pulmonary resection is reported in the literature as added value [12,13].

Video assisted thoracoscopic surgery (VATS) has been adopted in pediatric age for the treatment of CLM. Minimally invasive approach to the thorax in small children and infants needs to be provided by a super dedicated, trained team. The success of VATS in pediatrics largely depends on the surgeon’s ability to understand the airways, vascular system and lung parenchyma anatomy in CLM. To date no reports about the use of VR HMD in pediatric surgical clinical practice are available. VR HDM could promote a safe pulmonary resection in small children with critical anatomy. The aim of this report is to introduce a VR HMD model in VATS procedure to improve the quality of care in children with CLM.

## 2. Patients and Methods

### 2.1. Patients

A series of three patients, submitted to surgery for CLM in the Surgical Department of “Vittore Buzzi” Children’s Hospital by the same team, were studied. Demographic, clinical and pre-operative instrumental data, intervention and outcome were recorded. All patients underwent chest X-ray, computed tomography (CT) and/or magnetic resonance imaging (MRI) before surgery.

The study was retrospectively performed according to the Declaration of Helsinki. Informed written consent was obtained from the parents and/or legal guardian after receiving information about the study.

### 2.2. Three-Dimensional (3D) Models

To create a highly detailed 3D model of patient’s malformation, considering the size of anatomical structures at pediatric age, a 0.6 mm-thick CT scan in arterial and venous phases was acquired. CT images were then exported into DICOM files and loaded into 3D Slicer v.4.11 (https://www.slicer.org) [14], a free open-source software that allows image segmentation, i.e., the labelling of anatomical structures in medical images to separate them from the background and from each other. Given the complexity of the involved structures, a semiautomatic segmentation approach was used, combining the in-built region-growing and threshold algorithms with manual corrections. The segmentations were then used to obtain 3D surface models of the lung malformation and the different anatomical structures. These models were zoomable and viewable from many viewpoints. Moreover, in contrast with traditional volume-rendering techniques, each model can be hidden or shown in transparency independently from the others, which allows one, for example, to visualize inner vascularization or focus on specific structures.

The segmentations and the 3D scenes finally obtained were reviewed and approved by a radiologist for consistency with the source medical images.

### 2.3. Virtual Reality HMD Model

Using an in-house developed plugin for 3D Slicer, the obtained 3D models of patient’s malformation were loaded into a HMD through a Universal Serial Bus (USB) connection. Specifically, we employed the Oculus Quest v. 1 (META Inc., Menlo Park, CA, USA), an all-in-one HMD equipped with an OLED display with a resolution of 1440 × 1600 pixel per eye and a refresh rate of 72 Hz.

A previously developed app [15] readapted for Oculus Quest was used to provide the surgical team with an immersive visualization of the 3D reconstruction inside a dedicated Virtual Reality Environment (VRE).

During preoperative planning, the surgeons wore the HMD and, using its wireless controllers, were able to launch the app, choose a reconstruction and explore it. More specifically, the 3D models could be moved, rotated, zoomed and its components could be made transparent. Surgeon’s point-of-view could be shared with the rest of the team, using a computer equipped with a dedicated desktop app, to allow and encourage a fruitful discussion aimed at agreeing on the best surgical approach [16,17].

### 2.4. Preoperative Virtual Reality HMD Setup Evaluation

All the CT images were reviewed on the resection plans using the patient-specific 3D models visualized in the VRE. The images from CT scan and the 3D models visualized with the VR HMD were both oriented according to the patient positioning for VATS procedure. Navigation towards the critical anatomical structures (vessels and tracheobronchial tree) was obtained through the rotation of the images. Rotation and navigation through the malformation allowed one to evaluate the relationships among structures and to define the surgical steps including dissection and ligation. These data were postoperatively compared with the intraoperative findings.

### 2.5. Surgical Technique 

The VR workstation and the 3D reconstruction, displayed on a monitor, were located in the operative room (OR) to provide an additional review of the anatomy when required by the surgeon.

An experienced pediatric surgeon performed the 3D VATS lobectomy. Surgical assistance was provided by a resident physician.

Surgery was carried out under general anesthesia and continuous Erector Spinae Plane (ESP) block with cathether; the patient was positioned in lateral decubitus (contralateral to the side of the malformation). Single lung ventilation was instituted via Arndt Endobronchial Blocker (Cook^®^).

Trocar positioning included two ports on the mid axillary line to allow the camera exchange from bottom position to the apex; operative trocars were fixed respectively medially and laterally to the previous line.

## 3. Results

### 3.1. Patient’s Data

Three infants (2 females, 1 male) were included in the project. In all patients the malformations were prenatally detected (2 lobar sequestrations and 1 bronchogenic cyst). The prenatal and neonatal counseling were performed according to the diagnostic-therapeutic program as following: chest X-ray at birth, 2nd level imaging at 3–4 months of life. According to our Hospital protocol, a combined chest CT scan–MRI was routinely performed in the first month of life in case 1 and in the immediate preoperative time in patient n. 2 and n. 3.

At birth, respiratory distress was noted only in patient n.1. Surgery was performed at 4 months, 4 years and 3 years respectively. 

Clinical and demographic data of the patients are reported in Table 1.

### 3.2. Preoperative Planning

Each clinical case was discussed by a multi-disciplinary team (including neonatologists, anesthesiologists, radiologists, pneumologists, pathologists, pediatricians), to agree on the timing for treatment and the best surgical approach.

In the light of patient’s malformation, anatomy was reviewed inside the VRE. The 3D model was preoperatively examined by the surgical and anesthesiologic team to learn about anatomy and margin decision-making during MIS.

The use of preoperative Oculus Quest 3D reconstruction allowed the surgeon to explore and interact, in a virtual environment, with the scene while sharing with the team what one is viewing through the wireless-connected computer equipped with a dedicated software.

VATS was preoperatively simulated few days before operation in each patient and in presence of all the surgical team (surgeons, anesthetists and nurses). A re-do navigation was also retraced in the operative room (OR) just before surgery and during surgery, when needed [18].

Preoperative virtual navigation into the thorax permits an optimal study of the individual anatomy. Cystic location and surgical margin decision for resection during minimally invasive surgery was obtained.

In terms of simulation training, preoperative review of patients’ individual anatomy, malformation size, and malformation location is beneficial for assessment of surgical resectability.

### 3.3. Surgical Procedure

The operations were entirely VATS performed (right in 2 cases and left thoracoscopy in one case). All VR images resulted to be well assessable under the same orientation adopted for VATS, the lateral position. Preoperative navigation in lateral position to target the lung malformation and the pulmonary hilum showed a proper evaluation of the hilum and lung structures, arteries, veins and bronchus. The different orientations of view showed a better understanding of the relationship among the different bronchial structures, artery, and veins. A surprisingly clear anatomical view obtained was attributed to the high resolution images provided by Virtual Reality HMD set-up.

In all patients during critical dissection or prior to no-return decisions the surgeon returned to a vision replay in order to confirm the surgical maneuver to avoid errors such as stapling of bronchovascular structures or vascular structures whose closure could definitively exclude lung function.

**Figure 1:**
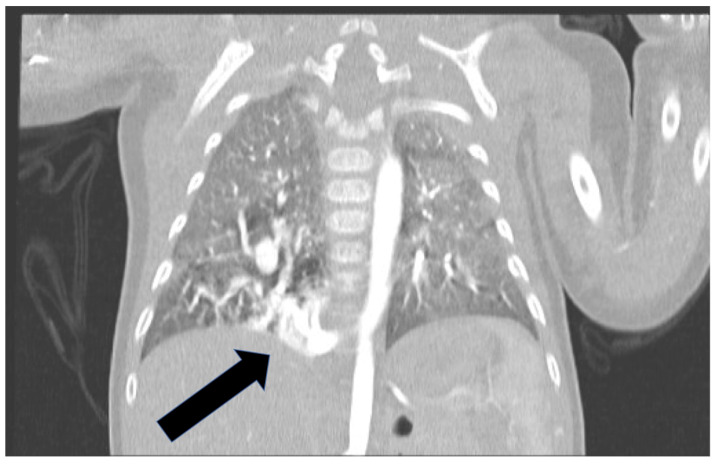
In patient 1, a very large branch of 6.5 mm in diameter (equal to abdominal aorta), originating from the right side of thoracic descending aorta and giving systemic arterial supply to the malformation was showed.

In patient 1, affected by pulmonary sequestration, the evaluation of a 3D model helped to learn the surgical approach and modality of dissection at hilum. In this patient a very large branch of 6.5 mm in diameter (equal to abdominal Aorta), was originating from the right side of thoracic descending Aorta, close to the diaphragm, giving systemic arterial supply to the malformation, Figure 1.

**Figure 2:**
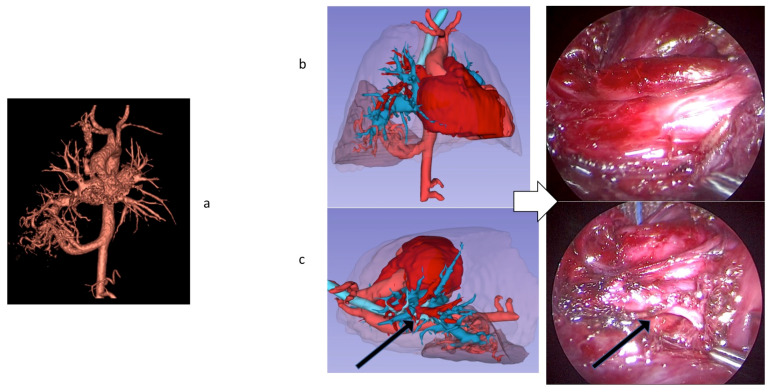
Malformation in patient 1. Panel (**a**)**,** anatomical details of sub-subsegmental pulmonary artery branches; Panel (**b**), the augmented virtual reality model allows the surgeon and all the team to preoperatively interact in the operative room. Panel (**c**), intraoperative findings: isolation of the inferior right pulmonary vein closed in its dilated portion, 3 venous vessels efferent from the lesion.

A 7 mm venous vessel leaving the malformation and draining into an ectasic right inferior pulmonary vein was detectable, therefore determining a left-to-left shunt, with left atrial overload. Intraoperatively, the sequestration in the right lower lobe, with an afferent vessel from supradiaphragmatic aorta, was confirmed. The high load vessel was ligated at its deflection from the aorta, followed by isolation of the inferior pulmonary lobe from the middle lobe; vascular and bronchial structures afferent to the lesion were closed and sectioned. Then the inferior right pulmonary vein was isolated and closed in its dilated portion, together with 3 venous vessels efferent from the lesion. The lobar sequestration was extracted and resulted to be about 40 ml of volume, Figure 2.

**Figure 3:**
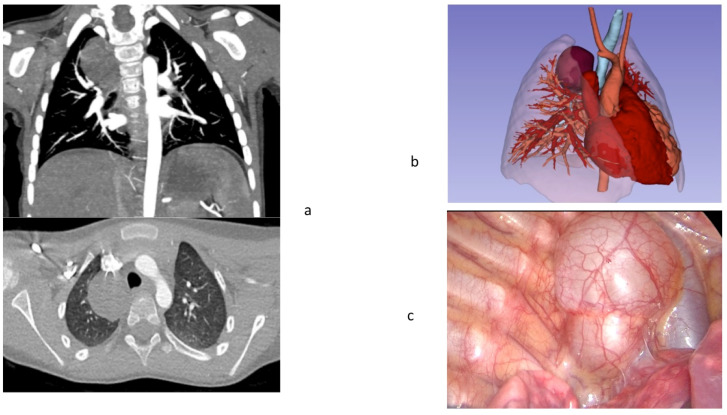
Malformation in patient 2. Panel **a**, Preoperative anatomy of bronchogenic cyst; Panel **b**, simulation of thoracoscopic approach; Panel b, Virtual Reality HMD set-up is equipped in the theatre just before proceeding surgery; Panel **c**, thoracoscopic view of the cyst.

VR HMD preoperative evaluation of the pulmonary arteries, veins or bronchus variants allowed for identification and easy dissection of the dilated veins close to the hilum. Artery and venous ligation and evaluation of its relation with bronchus was easily and securely performed. Preoperative evaluation of the lesion under images oriented in patient thoracoscopic position, the right lateral side, favored the approach to the arterial branch arising from the aorta in patients 1 and 3. Trocar positioning also had benefit from the preoperative VR HMD study in all cases. In patient 2, the bronchial dissection was carried out at hilum because of the dimension of the cyst; preoperative orientation of the patient on the lateral position was in favor of a safer dissection of the lesion and no damage of the bronchial tree was recorded, Figure 3.

Based on VR HMD, in all patients the correlation between intraoperative findings and preoperative reconstruction showed higher resolution than thoracoscopically expected. Details of segmental pulmonary artery branches and bronchus were well identified at hilum and made vascular and bronchial dissection easier. Subsegmental bronchi dissection on VR HMD turned out to bring no redeeming advantages to the VATS procedure.

No intraoperative nor postoperative complications occurred. Thoracic drains were not positioned and patients were discharged 48 hours postoperatively. No late complications have been documented.

Detailed visualization of anatomical structures using VATS and VR HMD is reported in Table 2.

Several advantages with the use of VR HMD setup comparing to preoperative conventional images were noticed and are summarized in Table 3.

### 3.4. Postoperative Navigation

Postoperatively, the surgeon analyzed the critical anatomy in all patients. Anatomical traps and surgical details were reviewed and compared to VATS recorded video. This was displayed to the operator and to the team in a relevant manner that consisted of appropriated orientation of the malformation in function of the patient positioning for VATS procedure. Postoperative navigation of the same scene in the VRE was an important educational tool for trainees. VR simulation gave the opportunity to analyze and teach technical aspects of the operation and offered the opportunity to re-view each procedure several times in order to define the most suitable surgical strategies and to avoid mistakes and errors.

## 4. Discussion

Virtual Reality HMD set-up for planning thoracoscopic surgery was performed in a series of pediatric patients with diagnosis of CLM. The preoperative VR HMD evaluation allowed for a navigation into the malformation with the aim to explore, interact, and make the surgeon more confident and skilled to answer to the traps [19].

Preoperative planning with interactive 3D CT reconstruction has been largely adopted in adult patients as a useful method to enhance the surgeon’s knowledge of the patient’s anatomic variations [10,11].

According to data from the literature in adult patients, intraoperative 3D guiding and 3D vision in VATS may contribute to the safety and accuracy of anatomic resection, VR HMD is described to be more helpful for VR-based segmentectomy planning tool in adults patients in case of pulmonary tumor resection. In adults, preoperative VR HMD navigation allows pulmonary resection, and essential changes of surgical strategy are reported in almost 40% of the cases [20]; segmentectomies were successfully performed and all the oncologic resections were adequately done in all cases preoperatively evaluated under VR navigation. These data suggest that the preoperative review of surgical strategy is suitable and it does not need more imaging from the consult of radiologists or imaging technicians.

Our preliminary experience demonstrates that VR HMD in children and infants is technically feasible and applicable to the clinical study of complex pulmonary anatomy in small thoracic space. It represents a beneficial consequence for the study of individual anatomy. The advantages of such technology are represented by the opportunity to share the difficult anatomic approach with the surgical team and revisit situations of surgical risk, trap errors and the way to avoid them. Each operator may become more expert and confident in congenital malformation management. The scene sharing with the surgical team, including the nurses, contributes to the best care and child safety for a better outcome and represents an opportunity for the surgical team building.

Moreover, the realistic in-depth perception is an interesting detail reported in the literature [21,22]. Preoperative VR HMD navigation represents a useful tool to gain a better and more realistic insight into a patient’s specific anatomy thanks to in-depth perception. The best feedback comes from pulmonary resection and subsegmental bronchial and vessel dissection. In our series, in-depth perception was clearly appreciated during vessel dissection at the hilum in patient 1.

Some limitations of such a technique in children are the lack of simulation of virtual pulmonary resection, and the consequent lack of lung inflation/deflation simulation, and pulmonary manipulation. VR reconstruction of subsegmental bronchi visualization was also poor due to the dimension of the structures on CT scan. In these cases pulmonary segmentectomy and lung sparing are more securely carried out under thoracoscopic view. The most secure VR HMD approach is the hilum in case of difficult and critical anatomy. Pulmonary segmentectomy planning, compared to adults, seems to be not equally feasible in small children.

To our knowledge, VR HMD model has not been previously described in pediatric VATS. VR HMD was an effective simulation of the surgical navigation to the malformation under thoracoscopic approach and represents a model to promote a safer VATS for the treatment of congenital malformations. The complexity of the anatomical surrounding vessels and vital organs could benefit from creation of 3D models. We chose HMD model because of its compact size, low price ($399–499, depending on the internal storage capacity) and features (e.g., allows hands-free interaction, especially useful in sterile contexts such as operating rooms, instead of using the supplied controllers). Developing VR HMD associated to the 3D modeling study could guarantee better clinical results, reducing complications and costs [23,24,25]. Greater safety in the conduct of procedures also allows better adherence to the principles of fast-track surgery, avoiding for example the positioning of drains, with better control of postoperative pain and a reduction in hospitalization times. Further studies on the use of VR in pediatric surgery could also lead to a reduction in surgical times, with a consequent improvement in the anesthetic and surgical outcome.

We acknowledge that the current study has some limitations in terms of a small number of patients. To draw definite conclusions to implement such technology, more detailed prospective, extensive, and comparative analyses are required. In our study the results on the operative times were not considered; however, an advantage compared to standard treatment could not be excluded; further studies are mandatory to define the surgical and anesthesiological outcomes related to operative duration. Moreover, in the next few years also pediatric patients will benefit from an Augmented Reality (AR) view as a virtual projection of anatomy in the surgical field to allow an AR-guided pulmonary resection also in small patients. AR could also be supported during more sophisticated minimally invasive surgical procedures such as robotic approach.

## 5. Conclusions

The treatment of CLM needs a standardization of care, a dedicated teamwork able to promote a personalized surgical planning and a culture of safety for surgery of lung congenital malformations in small patients. Simulation in preoperative study may assist surgeons intraoperatively to recognize, address and report errors and traps when they arise. Introduction of Virtual Reality HMD in Pediatric Surgery VATS allows the surgeon to be more confident in small thoracic spaces and promote a culture of safety and error trap monitoring. Such technology could help to bridge the gap in the treatment of a group of conditions that may be faced rarely also by experienced pediatric surgeons [20]. All the pediatric team may benefit of VR HDM in terms of amelioration of quality of care, and learning curve in supporting surgeon during VATS procedure. A development of surgical simulation models and a teaching program dedicated to education and training in minimally invasive surgery is suitable among the pediatric surgery community. Further studies should demonstrate all the benefits of such technology in children submitted to VATS procedure.

## Figures and Tables

**Table 1:** Patient’s data.

Features	Patient 1	Patient 2	Patient 3
Age at surgery	4 months	4 years	3 years
Sex	F	F	F
Gestational age	term	term	term
Type of malformation	Right lobar bronchopulmonary sequestration Anomalous venous return	Right bronchogenic cyst	Left extralobar bronchopulmonary sequestration
Prenatal diagnosis	20 weeks gestation	22 weeks gestation	No prenatal diagnosis
Preoperative imaging	Intralobar pulmonary sequestration with surrounding areas of air trapping, possible hybrid malformation microcystic CPAM-pulmonary sequestration	Large Right bronchogenic cyst	Left extralobar sequestration
Symptoms at birth	Respiratory distress	No symptoms	Recurrent bronchopneumonia
Histology	Intralobar pulmonary sequestration and cystic adenomatoid malformation type 2	Bronchogenic Cyst	Extralobar pulmonary sequestration and adenomatoid malformation type 3
Surgery	Right Thoracoscopic lower lobectomy Dissection at the hilum	Thoracoscopic excision of bronchogenic cyst, dissection at the hilum	Thoracoscopic excision of pulmonary sequestration

**Table 2:** Video-assisted thoracoscopic surgery (VATS) and Virtual Reality HMD set-up. Visualization of the anatomical structures.

Anatomical Structure	Virtual Reality HMD Set-Up	VATS
Visualization of hilum	-Excellent 3D view at the hilum of segmental structures	-2D = view
	-Opportunity of rotation and evaluation of critical anatomy under different views 360°	
Visualization of Bronchus and		
	-Excellent 3D view on segmental bronchi	-Poor orientation
-Segmental bronchi -Bronchi orientation	-Excellent 3D orientation and relationship among structures	-Poor view on the bronchi 2D
Vessels		
-Segmental artery and vein visualization	-Excellent 3d view on segmentation	-2D view
-Vessel orientation	-Excellent view on segmentation during orientation	
Approach to the pulmonary tissue	-realistic in-depth perception	-2D view, no depth perception
Pulmonary tissue	-No view on subsegmental structures	
	-No advantages on tissue sparing	-Good control on tissue resection and manipulation
	-No tissue manipulation	

VATS: video assisted thoracoscopic surgery.

**Table 3:** VR HDM usefulness in preoperative evaluation.

Advantages	Debatable
Vessel anatomy variants detection	Sub-segmental artery, veins, bronchi division
Segmental artery, veins, bronchi division	Lack of simulation of lung inflation/deflation
Arteries, veins, bronchi orientation	Lack of simulation of virtual resection/transection and manipulation of pulmonary tissue
Realistic in-depth perception	

## Data Availability

Data reported in this study are available upon request from the corresponding author.
